# South Dakota State Veterinary Medical Association

**Published:** 1900-11

**Authors:** J. P. Foster

**Affiliations:** Secretary


### SOUTH DAKOTA STATE VETERINARY MEDICAL ASSOCIATION.

IN response to a call issued by certain members of the profession, the veterinarians of South Dakota met at eight o'clock P.M., October 9, 1900, at the Depot Hotel, Huron, S. D., for the purpose of organizing a State Veterinary Medical Association.

The meeting was all that could have been desired, both in point of attendance and interest shown by those present in the movement. Dr. J. W. Elliott was elected temporary chairman and Dr. H. O. Sanford, temporary secretary, and Drs. Moore, Hicks, and Foster were appointed a committee to draw up by-laws to be approved by the members of the Association.

The by-laws having been presented and approved, election of officers took place, with the following result: President, J. W. Elliott, V.S., Aberdeen; Vice-President, D. A. Cormack, D.V.S., Watertown; Treasurer, H. O. Sanford, M.D.C., Beresford; Secretary, J. P. Foster, V.S., Selby. Drs.

Cormack, Hicks, and Moore were selected as a committee to frame suitable bills to be presented at the next session of the Legislature.

After discussing other matters of interest the meeting adjourned, to meet again in January, exact date and place to be decided upon by the President.

J. P. Foster,
Secretary.

